# Correction: Chen et al. Tellurium Doping Inducing Defect Passivation for Highly Effective Antimony Selenide Thin Film Solar Cell. *Nanomaterials* 2023, *13*, 1240

**DOI:** 10.3390/nano14221795

**Published:** 2024-11-08

**Authors:** Guojie Chen, Xiangye Li, Muhammad Abbas, Chen Fu, Zhenghua Su, Rong Tang, Shuo Chen, Ping Fan, Guangxing Liang

**Affiliations:** 1 Shenzhen Key Laboratory of Advanced Thin Films and Applications, Key Laboratory of Optoelectronic Devices and Systems of Ministry of Education and Guangdong Province, College of Physics and Optoelectronic Engineering, Shenzhen University, Shenzhen 518060, China; 2060451014@email.szu.edu.cn (G.C.); williamlixiangye@gmail.com (X.L.); abbas.cssp@hotmail.com (M.A.); zhsu@szu.edu.cn (Z.S.); fanping@szu.edu.cn (P.F.); lgx@szu.edu.cn (G.L.); 2 School of New Energy and Energy Conservation and Environmental Protection Engineering, Foshan Polytechnic, Foshan 528137, China; rongtang@fspt.edu.cn

In the original publication [1], there was a mistake in Figure 7 as published. The author regrets the existence of an error made during the proofreading process, in the currently published Figure 7b, showing the C-f-T data for Device-T2. Specifically, Figure 7a was duplicated in place of in Figure 7b by mistake. However, it is important to note that data shown in the original Figure 7d (showing the Ea of Device-T2) and Figure 7f (depicting the Nt of Device-T2) were estimated according to the corresponding realistic measurement C-f-T data (in the as-corrected Figure 7b). And the data in the original Figures 7c (showing the Ea of Device-T0) and 7e (showing the Nt of Device-T0) were estimated according to the corresponding realistic measurement C-f-T data (in Figure 7a). These adjustments have been made to ensure the fidelity of the data representation. The corrected Figure 7 is appears below:

The authors state that the scientific conclusions are unaffected. This correction was approved by the Academic Editor. The original publication has also been updated.

## Figures and Tables

**Figure 7:**
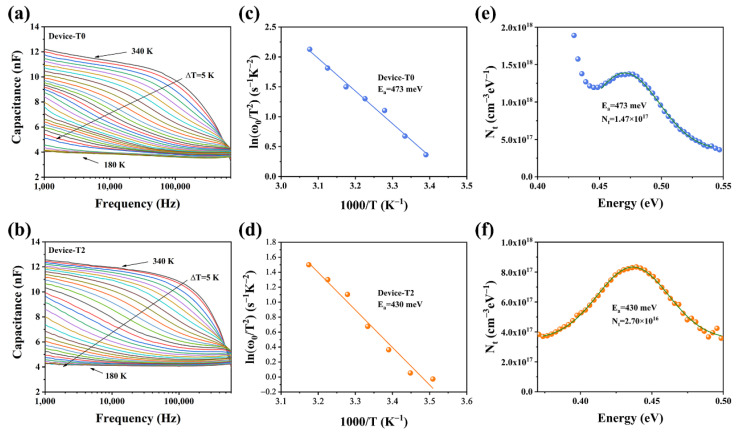
(**a**,**b**) Capacitance−frequency−temperature (C−f−T) spectra (different colored lines represent capacitance values at different temperatures) of the T0 and T2 devices, respectively; (**c**,**d**) Arrhenius plots of the T0 and T2 devices; and (**e**,**f**) defect densities of T0 and T2 devices, respectively.
